# Angioleiomyoma: An Update with a 142-Case Series

**DOI:** 10.3390/life14030338

**Published:** 2024-03-04

**Authors:** Mathilde Bernard, Louis-Romée Le Nail, Gonzague de Pinieux, Ramy Samargandi

**Affiliations:** 1 Service de Chirurgie Orthopédique et Traumatologique, CHRU Trousseau, Faculté de Médecine de Tours, Université de Tours, 1C Avenue de la République, 37170 Chambray-les-Tours, France; 2 CNRS ERL 7001 LNOx: Leukemic Niche & redOx Métabolisme, EA 7501 GICC, Université de Tours, 37000 Tours, France; 3 Service d’Anatomie et Cytologie Pathologique, CHRU Trousseau, Faculté de Médecine de Tours, Université de Tours, 1C Avenue de la République, 37170 Chambray-les-Tours, France; gonzague.dubouexic@univ-tours.fr; 4 Department of Orthopedic Surgery, Faculty of Medicine, University of Jeddah, Jeddah 23218, Saudi Arabia

**Keywords:** angioleiomyoma, leiomyoma, soft tissue tumors, MRI, caldesmon, dark reticular sign

## Abstract

Angioleiomyomas are uncommon, noncancerous, smooth muscle tumors that primarily arise from blood vessels. Previous studies have yielded limited data due to the lack of interdisciplinary approaches or restricted patient pools. This study aims to provide a comprehensive analysis of angioleiomyomas, including the demographic, clinical, radiological, and histopathological features, with a large number of patients. Conducted as a retrospective investigation at a single center from January 2005 to June 2023, this study involved 142 patients. Relevant information was extracted from electronic medical records, covering clinical, radiological, histological, and demographic details. Angioleiomyomas mostly occurred at age 59 (1–87), predominately affect females (53%) and commonly arise in subcutaneous tissue (85%) and the lower limbs (76%). MRI findings revealed characteristic signals, with a high prevalence of the solid histologic type (65%), often displaying a reticular sign. Smooth muscle Actin was universally present (n = 95/95), while Desmin and Caldesmon showed positive expression in 83% (n = 71/85) and 98% (n = 92/94) of cases, respectively. This study presents an updated and comprehensive analysis of angioleiomyomas. Typically appearing as well-defined nodules in the extremities, these tumors can be effectively diagnosed using MRI, though histopathological analysis is generally essential for confirmation. Treatment primarily involves straightforward excision, with notable low complication and recurrence rates.

## 1. Introduction

Angioleiomyomas are benign smooth muscle tumors primarily originating from blood vessels, specifically the tunica media of small to medium-sized arteries and arterioles. They commonly affect individuals aged between the third and fifth decades of life, with a higher prevalence in females than males [1]. These tumors exhibit distinct histopathological characteristics and clinical presentations, emphasizing the importance of their accurate recognition and understanding for precise diagnosis and appropriate management.

Clinically, angioleiomyomas typically present as solitary, well-defined, gradually enlarging masses. They frequently elicit pain or tenderness upon palpation and predominantly emerge in the skin and subcutaneous tissues, notably in the upper [2,3,4,5,6,7] and lower limbs [8,9,10]. However, occurrences in other anatomical sites, including the trunk, head, and neck [11,12], and even intra-abdominally [13], have been documented. The precise cause of angioleiomyomas remains unclear.

Histologically, angioleiomyomas display a distinctive pattern characterized by bundles of spindle-shaped smooth muscle cells arranged around numerous small vascular spaces [9]. These tumor cells express smooth muscle Actin and Desmin, confirming their origin from smooth muscle. Immunohistochemical analysis proves valuable in distinguishing angioleiomyomas from other vascular tumors and neoplasms with smooth muscle differentiation [11].

Previous studies have explored certain connections between clinical aspects, localization, and histology, but the results were contradictory [14,15]. Furthermore, previous published studies had a limited number of patients evaluated with clinical, radiologic, and histologic analysis, often confined to case series or case reports. To the best of our knowledge, there is a dearth of studies incorporating a substantial number of patients. The most extensive study, comprising 562 patients, was published in 1984 [14]. While it stands as the largest study with pathologically proven angioleiomyoma, it is notable that radiological imaging, including radiographs, ultrasound, and Magnetic Resonance Imaging (MRI) features, was not assessed.

The objective of this study is to offer a recent and thorough update on angioleiomyoma. We aspire to accomplish this goal by presenting insights from one of the largest series, covering demographic, clinical, radiological, and histopathological features. Through this comprehensive exploration, we seek to enhance the understanding of this rare lesion. Such improved comprehension can empower healthcare professionals to effectively diagnose and manage these tumors.

## 2. Materials and Methods

### 2.1. Study Design

This descriptive observational study, constituting a retrospective cohort analysis, sought to investigate the records of 142 patients diagnosed with angioleiomyoma at our institution from January 2005 to June 2023. Our institution serves as a tertiary referral center for bone and soft tissue tumors. Ethical approval for this study was obtained from the institutional review board (approval number: 2023_086).

### 2.2. Data Collection

The medical records of patients diagnosed with angioleiomyoma were retrieved from our institution’s electronic database. The outcome measures encompassed demographic data, clinical information, radiological reports, histopathological results, and details regarding treatment.

### 2.3. Inclusion and Exclusion Criteria

Patients were considered for inclusion in the study if they had a confirmed diagnosis of angioleiomyoma based on histopathological examination (n = 142). Cases diagnosed between 2005 and 2023 were included to ensure a sufficient sample size and up-to-date data. Among the 142 included patients, 82 had accessible MRI, 56 had accessible ultrasound, 23 had accessible radiography, and 60 lacked imaging data but were still included in the demographic and histological analyses.

The exclusion criteria involved patients without a confirmed anatomopathological diagnosis and those with a concurrent diagnosis of other vascular tumors or neoplasms.

### 2.4. Data Analysis

Descriptive statistics were employed to summarize the demographic characteristics of the study population, encompassing age, gender distribution, and the anatomical sites affected by angioleiomyoma. Qualitative variables were analyzed using frequencies and percentages, while quantitative variables were assessed using means and ranges. Clinical presentations, including pain, tenderness, and other associated symptoms, were examined. Radiological reports were scrutinized to ascertain the imaging characteristics of the tumors, including the presence or absence of a reticular sign (MRI). This sign was defined as the presence of a dark reticular image within the lesion on T2-weighted sequences [1,16].

The histopathological findings, including smooth muscle Actin, h-Caldesmon, and Desmin staining results, were meticulously documented. The histopathological types were categorized according to Morimoto’s stages, which delineate three distinct mass categories: venous, cavernous, and solid, based on the characteristics of vessels and muscle [17].

The treatment modalities, such as surgical excision or conservative management, were recorded. The rate of recurrence or complications, if available, was also subjected to analysis. Demographic data were presented with the minimum and maximum range. To assess the correlations suggested by previous studies, statistical tests (Fisher’s exact test and the chi-squared test) were performed on the demographic, clinical, and histopathological data. A significance level of *p* < 0.05 was selected to establish statistical significance.

## 3. Results

The demographic characteristics of the study population revealed a mean age of 59 (range: 51–69), with a slightly higher prevalence in females (53%, n = 75) compared to males (47%, n = 67). The women-to-men ratio was 1.1/1.

Angioleiomyomas most commonly affected the extremities, notably the lower limbs (76%, n = 108) and upper limbs (21%, n = 30). A smaller proportion involved the head (3%, n = 4), with none affecting the trunk. Among the lower limb cases (n = 108), the most prevalent locations were the foot (30%, n = 18), knee (28%, n = 30), and leg (21%, n = 23). In the upper limb cases (n = 30), the predominant locations were the hand (73%, n = 22), elbow (20%, n = 6), and arm (7%, n = 2), respectively.

A cross-analysis of gender (male/female) versus location (upper limb, lower limb, head and neck) using Fisher’s exact test yielded a *p*-value of 0.35, indicating that the result is not statistically significant (Table 1).

### 3.1. Clinical Data

The mean size of the tumors, measured directly on the MRI in terms of the largest diameter, was 16.1 mm (range: 9.25–20). The majority of tumors were superficial, accounting for 92% (cutaneous: n = 7, subcutaneous: n = 120), while 8% were deep (subaponeurotic: n = 11).

The clinical presentations varied among the patients, with approximately 44% experiencing spontaneous pain or tenderness in the affected area (n = 63). Other associated symptoms, such as paresthesia, were reported in only one case. The lesion was predominantly discovered due to pain (44%, n = 63) or tumor growth (32%, n = 45). These data are summarized in Table 2.

Upon cross-referencing the clinical data (type of pain) with the histological data (Morimoto stage), no significant link between pain and a specific histological type was identified (Table 3).

**Figure 1:**
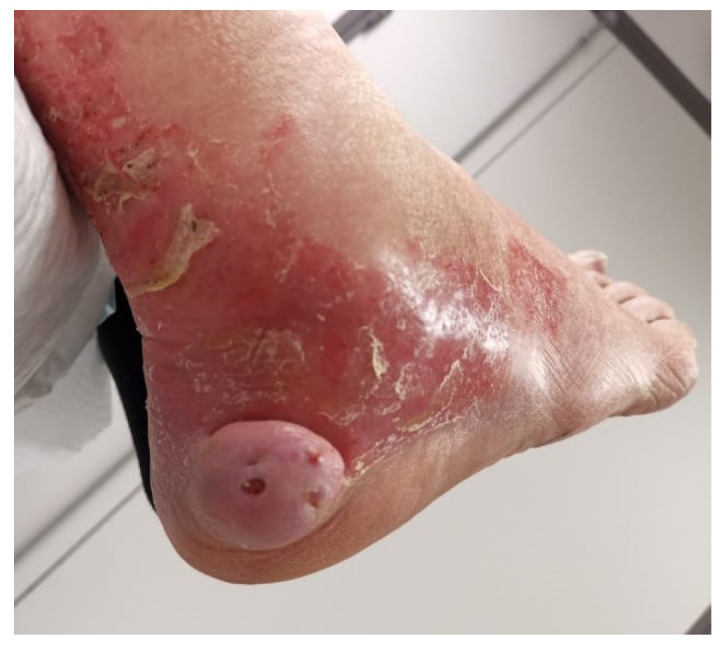
Man of 78 years old with atypical presentation of angioleiomyoma of the foot.

One of our cases exhibited clinical atypia, presenting as an inflammatory, dewy, and ulcerated mass, distinct from the typical characteristics described above (Figure 1).

### 3.2. Radiological Data

**Figure 2:**
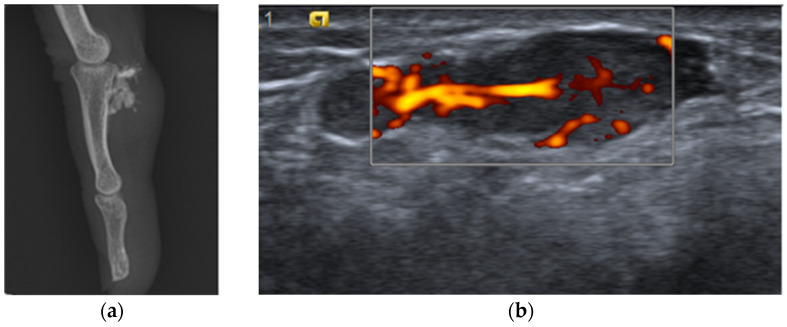
Radiological and ultrasound characteristics: (**a**) X-ray showing calcification of the lesion, and (**b**) ultrasound showing a well-circumscribed lesion hyperechogenic with hypervascularity on Doppler.

The radiological reports provided valuable insights into the imaging characteristics of the angioleiomyomas, which typically appeared as well-defined, round, or ovoid masses with variable vascularity on ultrasound. Figure 2 illustrates the ultrasound appearance of a hypervascularized angioleiomyoma and an angioleiomyoma with calcifications on radiography. In our study, only 14 out of 82 patients (17%) with available imaging demonstrated visible calcifications.

**Figure 3:**
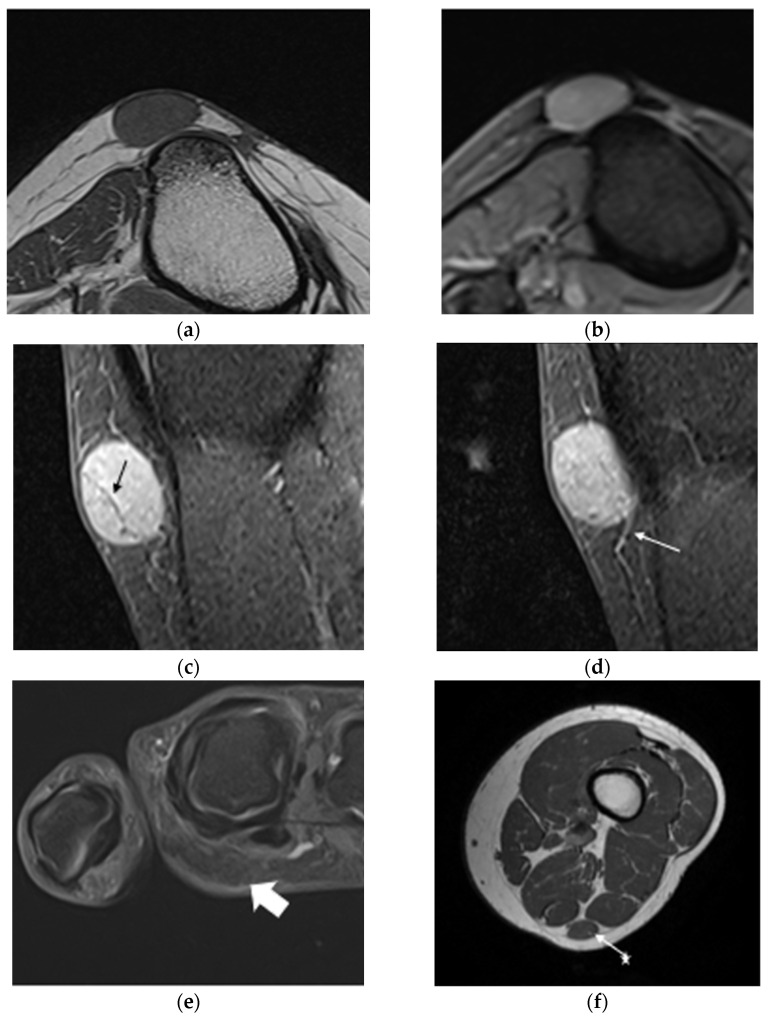
Magnetic resonance imaging characteristic of angioleiomyoma: common appearance of angioleiomyomas in a 50-year-old woman (**a**–**d**). (**a**) Axial T1-weighted MRI image of the left leg, demonstrating the subcutaneous low signal intensity of the lesion with well-defined low signal intensity margins located near the anterior tibial tuberosity but not attached to the periosteum. (**b**) Axial T2 gradient-echo sequences image showing a high signal intensity. (**c**) Sagittal fat-suppressed gadolinium T1-weighted image showing high contrast uptake with a reticular sign (black arrow). (**d**) The same sequence and cut plane as (**c**) but further cut showing high contrast uptake with afferent vascularization (white arrow). (**e**) Atypical presentation of axial T2-weighted fat-saturated image showing an iso-signal intensity (n = 3/78) located in the palmar region of the index finger of the right hand (white arrow). (**f**) Axial T1-weighted, subaponeurotic iso-signal lesion located between the semitendinosus and biceps femoris muscles of the left thigh (white arrow).

MRI revealed iso-signal intensity in T1 and high signal intensity in T2, with gadolinium uptake in 95% of MRIs (n = 79/82). Iso-signal T2 was identified in 3 cases (4%) out of 78. Figure 3 illustrates MRI characteristics with a reticular sign, hypointense peripheral zone, and vascularization. The reticular sign was observed in 56% of analyzed MRIs (n = 45/80). Table 4 presents an analysis of the presence of reticular signs according to the Morimoto stage (*p* = 0.52, Fisher’s exact test). Calcifications were found in the imaging in 17% of the analyzed MRIs (n = 14/83).

### 3.3. Histopathological Data

**Figure 4:**
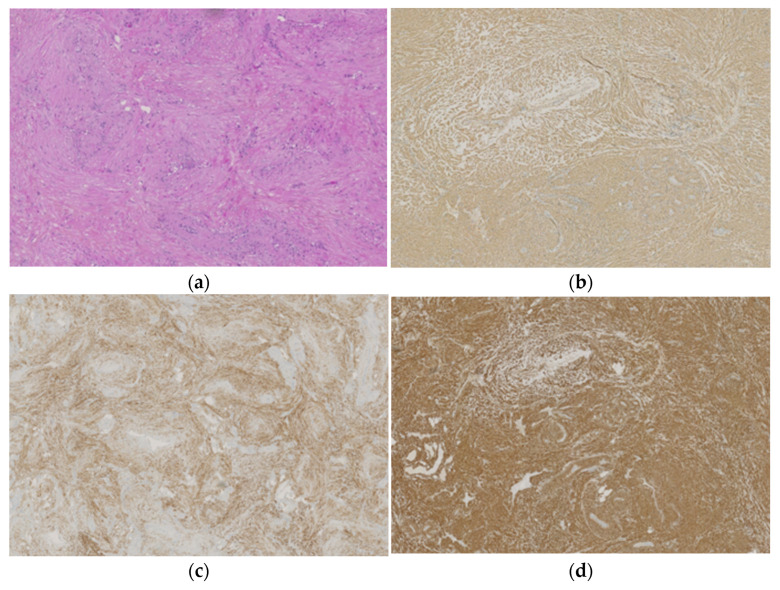
Histopathological features–magnification ×100. (**a**) Description on H&E staining: angioleiomyoma associating a proliferation of bundle-forming smooth muscle cells and vascular channels with narrow slit-like lumens, (**b**) Actin staining, (**c**) Desmin staining, and (**d**) Caldesmon staining.

**Figure 5:**
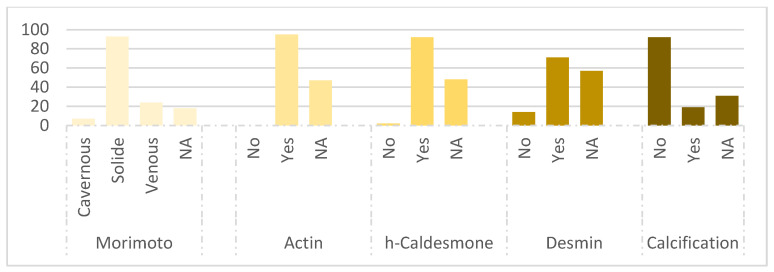
Distribution of patients (percentage) according to the Morimoto grade and immunohistochemical data (n = 142). NA = non analyzed.

Histopathological examination confirmed the diagnosis of angioleiomyoma in all cases, revealing a consistent pattern characterized by bundles of spindle-shaped smooth muscle fibers and vascular channels surrounded by thin capsules (Figure 4). The global results for the Morimoto type and immunohistochemical data are presented in Figure 5. Smooth muscle Actin was present in 100% of the cases analyzed (n = 95/95), while Desmin was positive in 83% of the cases (n = 71/85). Caldesmon was positive in 98% of the cases (n = 92/94). Calcifications were found in 19 cases (13%), and fat cells, thrombi, or lymphocytes were found in 21% of the cases (n = 30). The Morimoto types were classified based on the descriptions provided in the histopathological reports. Out of the analyzed cases, 65% were classified as solid (n = 93), 17% as venous (n = 24), 5% as cavernous (n = 7), and 13% remained unclassified due to a lack of description in the histopathology reports (n = 18). Importantly, no cases of malignant transformation or atypical features were observed.

Regarding treatment, surgical excision emerged as the primary modality, with 117 patients undergoing complete tumor resection. However, conservative management without surgical intervention was chosen in a minority of cases, constituting 17% (n = 25), particularly in patients with asymptomatic or small lesions. Among the operated patients, follow-up data were mostly unavailable. Notably, no follow-up was recommended after the excision of this type of tumor. No complications related to the treatment were observed. One patient sought medical attention for a second time, likely due to tumor recurrence, without any evidence of malignant transformation. Initially, the patient had been treated for leiomyoma, but anatomopathological reports were unavailable. The patient returned 19 years later with a lesion in the same location, ultimately diagnosed as an angioleiomyoma.

## 4. Discussion

### 4.1. Cross-Analysis

Previous studies on angioleiomyomas have often involved relatively few cases, except for the study by Hashisuga et al., which included 542 patients [14]. The study by Woo et al., with 16 patients, primarily focused on histology and demographic data [15]. They reported an equivalent prevalence in both men and women, aligning with our results. From a histological perspective, they did not find any correlation between clinical presentation and histology. In contrast, Hashisuga et al. [14] found that pain was correlated with the “solid” type in the Morimoto classification. However, the results of our study, as presented in the graph (Table 3), did not show any histoclinical correlation.

The study by Hashisuga et al. [14], involving 542 patients, holds the record for the largest number of inclusions to date. However, their data primarily encompass clinical, demographic, and anatomopathological aspects, without incorporating imaging data. In contrast, our study, featuring 142 inclusions and including clinical, demographic, and anatomopathological data, along with 82 cases having available MRI, provides a fairly comprehensive description of this tumor for a significant number of patients.

In their study, Hashisuga et al. [14] identified an association between clinical presentation, localization, and histology. They observed a male predominance for tumors located in the head and a female predominance for tumors in the upper and lower limbs. Additionally, they noted that pain was more predominant in the “solid” types of the Morimoto classification.

In the study by Edo et al. [16], where they examined 25 MRIs of patients with angioleiomyoma, a significant association was found between the reticular sign and histological subtype. The sign was more prevalent in the venous and cavernous subtypes. In our study, which had a larger sample size (n = 76), the reticular sign was more frequently observed (n = 32, 56%) in the solid subtype (n = 57), although these results did not reach statistical significance (*p* = 0.52).

In our study, there was a notable predominance of women (3/4) with head involvement and lower limb involvement (n = 59/108), while men had more frequent involvement of the lower limbs (n = 17/30). However, this difference did not reach statistical significance (*p* = 0.35). Additionally, we did not observe a clear predominance of pain in the solid types compared to the other histological types (*p* = 0.14). The distribution of the solid type was 65 to 80% in each clinical category, including spontaneous pain, pain upon palpation, paresthesia, and absence of pain.

Nevertheless, the proportion of different histological types described by Morimoto in the study closely aligns with our results. They reported 66% for the solid type (vs. 65% in our study), 23% for the venous type (vs. 17%), and 11% for the cavernous type (vs. 5%).

### 4.2. Imaging Features

Several studies have reviewed the imaging criteria supportive of angioleiomyoma. The study by Yoo [18], involving eight patients, demonstrates an iso-signal in T1, hyper-signal in T2, and enhancement after gadolinium injection, more or less homogeneously. The study by Alta [10], describing 26 lesions on MRI, corroborates these radiological characteristics. Our results are consistent with the existing literature. Two studies observe the presence of a dark reticular image within the lesion on T2-weighted sequences [1,16]. Edo et al. note the prevalence of this sign in the cavernous and venous types, which does not align with our findings.

When cross-referencing the presence of the reticular sign with the Morimoto stage, there is a tendency for the venous type to exhibit this sign, while the cavernous type almost does not (n = 1). The solid type, being the most common, presents it indiscriminately. However, these results did not reach statistical significance (Table 4) in our study.

In terms of the main radiological differential diagnoses, conditions such as neurogenic tumors, cystic tumors, glomus tumors, giant cell tumors of the tendon sheath, myxofibrosarcoma, and synovial sarcoma are noteworthy [2,3,10,19]. Similar to angioleiomyomas, schwannomas appear as a low or iso-signal intensity on T1, high signal intensity on T2, and show enhancement with gadolinium on MRI. However, vascular structures are typically not present in schwannomas. While the radiological characteristics may be quite similar, schwannomas are often located deeper and in proximity to a nerve. Clinically, the presence of a positive Tinel sign upon percussion of the tumor is commonly found with schwannomas, and this sign has high sensitivity for diagnosing schwannomas [20,21]. However, in cases where the mass is shallow, painful, and distant from major nerve pathways, differentiation before histology can sometimes be challenging.

Cystic tumors typically exhibit low signal intensity on T1, high signal intensity on T2, but do not show positive enhancement with gadolinium, and demonstrate signal loss with the FLAIR sequence.

The consideration of glomus tumors as a potential differential diagnosis for angioleiomyoma is warranted due to certain similarities in the clinical and radiological features, especially when they affect the hands and feet. However, it is crucial to note that while both glomus tumors and angioleiomyomas can present as painful masses, there are clinical distinctions. Glomus tumors tend to predominantly occur in a younger patient population and often present as a red–blueish nodule, typically in the subungual and hand regions. The pain associated with glomus tumors is generally more severe than that of angioleiomyomas and is often characterized by cold intolerance. Specific tests, such as the Love test or Hildreth test, can be valuable tools for diagnosing glomus tumors. Additionally, glomus tumors may be associated with bony erosion, particularly when they involve the distal phalanx, a presentation feature not commonly seen in angioleiomyomas [22].

Vascular malformations should be considered in the differential diagnosis of angioleiomyoma, as they can present with a painful mass that is typically compressible and pulsatile. These characteristics can be assessed during a physical examination or ultrasound examination. On imaging studies such as ultrasound or MRI, vascular malformations often display a serpiginous pattern due to their abnormal vascular channels and flow dynamics, and they may appear hypervascular on color Doppler. The presence of phleboliths is another distinctive characteristic of vascular malformations [23].

Giant cell tumors of the tendon sheath demonstrate an iso-intense signal on T1, high signal intensity on T2, but typically exhibit a multilobular appearance. They often occur adjacent to tendons, which distinguishes them from angioleiomyomas, as the latter are usually unilobular and well-defined. This distinction is important for the differential diagnosis, particularly in extremities such as the hands and feet [24].

Myxofibrosarcoma is another consideration, although it presents with a markedly distinct clinical profile characterized by an inflammatory and painful skin lesion. Myxofibrosarcoma occurs within the same age group and can exhibit uniformly low or iso-signal intensity on T1-weighted sequences with a superficial localization. However, it typically presents with high signal intensity on T2-weighted sequences and contains a heterogeneous myxoid component. The infiltrative pattern, often referred to as the “tail sign”, observed in myxofibrosarcoma can serve as a valuable diagnostic feature not observed in angioleiomyoma. It also primarily appears on the trunk, but localization on the limbs remains possible [25,26].

Synovial sarcoma is a crucial differential diagnosis to consider for angioleiomyoma, especially as it is more frequently located in the lower extremities, near the knee joint and foot and ankle [27]. MRI typically reveals iso-signal intensity on T1, high signal intensity on T2, and a predominantly heterogeneous lesion [28], which aligns with the characteristics of angioleiomyoma. Additionally, hemorrhage, necrosis, or calcifications within the lesion are common features of synovial sarcoma [27,29,30].

While large tumors may present with characteristics that make differentiation more straightforward, in the case of small, well-encapsulated synovial sarcomas, the appearance can be similar to angioleiomyoma. Therefore, a biopsy is often necessary to determine the malignancy of the lesion. In the study by Yoo [18], no calcification, necrosis, or hemorrhage was found in the observed angioleiomyomas. However, the study by Koga [1] mentions unusual calcifications, more commonly present in acral regions. In our series, 17% of lesions showed partial calcifications seen on imaging. In cases of uncertainty, performing a biopsy seems prudent.

### 4.3. Pathological Features

Some atypical cases of angioleiomyoma have been described in the literature, including ocular localization [12], extensive calcifications [31], and malignant transformation [32]. The malignant transformation involved the recurrence of angioleiomyoma with rapid growth, and the anatomopathological findings revealed an angioleiomyoma with a high proliferation rate. In our study, the proliferation index was consistently low, and we did not encounter any cases of malignant transformation. While two cases of recurrence were observed, the anatomopathological results favored a recurrence of benign angioleiomyoma.

The immunohistochemical study aimed to detect the presence of Actin, Desmin, and Caldesmon, which were the three main elements studied. Caldesmon is a muscle protein that regulates the contraction of smooth muscle [33]. It acts in conjunction with tropomyosin to control the interaction between myosin and Actin. Desmin is a protein present in smooth muscle and is often used as a marker in the diagnosis of angioleiomyoma. It is sought after to differentiate angioleiomyoma from myopericytoma and myofibroma, for which it is negative [11].

In our study, Actin was consistently present in the tumor. Caldesmon was present in 98% of cases examined, and Desmin was present in 83% of cases examined. Notably, when Desmin was negative, Caldesmon and Actin were positive. Based on the results of our study, there is a consideration whether the detection of Caldesmon could be more sensitive than the conventional examination of Desmin. Further research on the sensitivity and specificity of these markers could prove to be interesting.

### 4.4. Strength and Limitations of the Study

This study has certain limitations that should be acknowledged. The inclusion of cases from the anatomopathological database of the institution and the retrospective nature of the study led to some missing data. Incomplete information in the anatomopathological reports, lack of standardization in the immunohistochemical requests, and imprecise descriptions at times hindered the ability to precisely categorize the Morimoto classification stage. Some lesions underwent excision before further examination, driven by clinical suspicion of a benign nature. The data collection revealed the involvement of three specialties in the care of these patients before the final diagnosis: dermatologists, plastic surgeons, and orthopedic surgeons (with maxillofacial surgeons involved in three cephalic lesions). Seventy-nine cases were presented in a multidisciplinary onco-orthopedic consultation meeting, and all of these cases had available imaging.

Despite the limitations mentioned, the clinical data were well-described, with detailed information available from the anatomopathological reports, physician consultations, and the tumor board meetings where most patient cases were discussed before biopsy or excision. The standardized computerization of patient records ensured no issues with demographic data.

A notable strength of this study is its contribution to the literature with the largest number of patients, offering a comprehensive analysis of the demographic, clinical, radiological, and histopathological aspects of angioleiomyoma.

## 5. Conclusions

Our study contributes valuable insights into the clinical characteristics, radiological features, histopathological findings, and treatment outcomes of angioleiomyoma within our patient population. This tumor typically manifests as a well-defined nodule in the extremities. While MRI can be a useful diagnostic tool, histopathological analysis is generally necessary for confirmation. Additionally, the assessment of the Caldesmon levels may be of particular interest. The primary mode of treatment involves simple excision, demonstrating exceptional complication and recurrence rates.

## Figures and Tables

**Table 1:** Correlation between gender and location of angioleiomyoma (n = 142).

	Women n = 75 (53%)	Men n = 67 (47%)	Total n = 142 (100%)	*p* Value
**Upper Limb**	**13 (43%)**	**17(57%)**	**30(21%)**	**0.24**
Hand	9	13	22	0.22
Elbow	3	3	6	1.00
Arm	1	1	2	1.00
**Lower Limb**	**59 (55%)**	**49 (45%)**	**108 (76%)**	**0.44**
Toes	1	1	2	1.0
Foot	10	8	18	0.80
Heel	8	6	14	0.73
Ankle	9	8	17	0.99
Leg	15	8	23	0.19
Knee	14	16	30	0.44
Thigh	2	2	4	1.00
**Head and neck**	**3 (75%)**	**1 (15%)**	**4 (3%)**	**0.62**
Helix	0	1	1	0.47
Cheek	1	0	1	1.00
Lip	2	0	2	0.49

**Table 2:** Demographical and clinical characteristics according to patients (n = 142).

	n	%
**Gender**		
Female	75	53
Male	67	47
**Age**	Mean (min–max)	-
Total	59 (1–87)	-
Women	61 (32–87)	-
Men	57 (1–83)	-
**Mode of discovery**	n	%
Volume increase	45	32
Pain	46	32
Incidental finding on imaging	4	3
Discomfort	18	13
Inflammation	1	1
Presence of mass	18	13
No information	10	7
Type of pain *		
No pain	68	48
Tenderness	50	35
Spontaneous	13	9
Paresthesia	1	1
No information	14	10
**Laterality**		
Right	73	51
Left	66	46
No information	3	2
**Depth (n = 138)**		
Subcutaneous	120	85
Subaponeurotic	11	8
Cutaneous	7	5

* Several possible answers.

**Table 3:** Correlation between clinical symptoms and Morimoto classification (n = 131).

	Cavernous n = 7 (5%)	Solid n = 86 (65%)	Venous n = 21 (17%)	NA n = 17 (13%)	Total n = 131	*p* Value Fisher Test
No pain	4	42	16	5	67	0.06
Palpation pain	1	34	5	10	50	0.23
Spontaneous pain	2	9	0	2	13	0.07
Paresthesia	0	1	0	0	1	1

NA: not analyzed (no description in the histological report).

**Table 4:** Correlation between reticular sign and Morimoto classification (n = 76).

	Cavernous (n = 4)	Solid (n = 57)	Venous (n = 15)	*p* Value **
Reticular sign + (n = 42 *)	1	32	9	0.52
Reticular sign − (n = 34 *)	3	25	6	

* Data were collected from patients who had analyzable imaging and analyzable Morimoto stage, totaling 76 cases. ** A Fisher’s exact test was performed based on these data (*p* = 0.52).

## Data Availability

The data presented in this study are available on request from the corresponding author. The data are not publicly available due to privacy and ethical reasons.
